# A multidevice and multimodal dataset for human energy expenditure estimation using wearable devices

**DOI:** 10.1038/s41597-022-01643-5

**Published:** 2022-09-01

**Authors:** Shkurta Gashi, Chulhong Min, Alessandro Montanari, Silvia Santini, Fahim Kawsar

**Affiliations:** 1 Università della Svizzera italiana (USI), Faculty of Informatics, Lugano, Switzerland; 2 Nokia Bell Labs, Pervasive Systems, Cambridge, United Kingdom; 3 University of Glasgow, School of Computing Science, Glasgow, United Kingdom

**Keywords:** Biomarkers, Research data, Weight management

## Abstract

We present a multi-device and multi-modal dataset, called *WEEE*, collected from 17 participants while they were performing different physical activities. WEEE contains: (1) *sensor data* collected using seven wearable devices placed on four body locations (head, ear, chest, and wrist); (2) *respiratory data* collected with an indirect calorimeter serving as ground-truth information; (3) *demographics* and *body composition data* (e.g., fat percentage); (4) *intensity level* and *type* of *physical activities*, along with their corresponding metabolic equivalent of task (MET) values; and (5) answers to *questionnaires* about participants’ physical activity level, diet, stress and sleep. Thanks to the diversity of sensors and body locations, we believe that the dataset will enable the development of novel human energy expenditure (EE) estimation techniques for a diverse set of application scenarios. EE refers to the amount of energy an individual uses to maintain body functions and as a result of physical activity. A reliable estimate of people’s EE thus enables computing systems to make inferences about users’ physical activity and help them promoting a healthier lifestyle.

## Abstract (summary)

**Table:** 

Measurement(s)	Heart rate • Accelerometer • Oxygen Consumption • Gyroscope • Respiratory Rate
Technology Type(s)	PPG/ECG sensors • Accelerometer sensors • VO2 Master Analyzer • Gyroscope sensors • Wahoo ticker
Sample Characteristic - Organism	Healthy subjects
Sample Characteristic - Environment	Laboratory setting

## Background & Summary

Human energy expenditure (EE) refers to the *amount of energy an individual uses to maintain essential body functions (respiration, circulation, digestion) and as a result of physical activity*^1^. Knowledge regarding the expended energy or calories could help people (e.g., athletes, obese, diabetic) to plan their physical activity for leading a healthier lifestyle^2^. Additionally, it could be used to enable nutrition coaching for weight management purposes^3^. Devising methods for EE estimation (EEE) is a key enabler of the mentioned intervention strategies and it is the core goal of the dataset presented in this paper.

The gold-standard EE measurement methods are *direct calorimetry*–which measures body heat while the subject is inside a chamber–, *indirect calorimetry*–that consists of a mouth piece worn for respiratory gases analysis–and *doubly labeled water*–which measures carbon dioxide production during the interval between first and last body water samples^3–5^. Such techniques require the use of cumbersome and expensive equipment and are not feasible to measure EE in free-living conditions for specific activities on a minute by minute basis. Measuring EE in real-world scenarios in a fine-grained manner would enable obtaining valuable information regarding people’s physical activity and providing personalized and timely recommendations.

Considering the cost and practical limitations of gold-standard methods combined with the proliferation of ubiquitous computing^3^, several researchers started exploring the use of mobile and wearable devices for EEE^6–8^. Such devices are suitable for continuous monitoring of EE because they are unobtrusive and do not hamper the natural behavior of the user in free-living conditions. Additionally, they have the potential to provide a cheap and reliable solution to this problem. Despite the considerable research progress in sensor-based EEE, several challenges remain open. In particular, it is not evident which type of sensor, body position or combination thereof would enable reliable EEE. Also, there is a lack of studies investigating the quality of data and how it influences the robustness of EEE. Such investigations are impeded by the lack of sensor-diverse, multimodal and publicly available datasets, which could potentially enable the development of more accurate EEE techniques^4,7^. While there exist commercial wearable devices that measure EE (mainly using demographics data and accelerometer sensor), it is not clear how they compare to gold-standard measurements (e.g., indirect calorimetry) and new sensor-based techniques (e.g., physiological sensors).

To overcome such barriers and foster further developments in EEE, in this paper, we introduce a new, multimodal dataset collected from 17 participants using 7 wearable devices, each containing multiple sensors. The goal of the dataset is to enable the design and development of new sensor-based EEE techniques during rest and physical activity. To this goal, we design and run a data collection protocol, which consists of three activities, such as *resting*, *cycling* and *running*, each performed for 10 minutes. We picked these activities because they involve movements of different intensity levels (e.g., light, moderate and vigorous). In addition, they require full-, half- or no-body movement, which are representative of physical activities performed in everyday life, as discussed in^3^. Each physical activity was performed at two intensity levels to cover a wider range of movement intensity and explore the EE changes during such intensities. For instance, participants ran at two different speeds for 5 minutes each.

The dataset is collected using an indirect calorimeter, a headband, earbuds, two chest-belts (a commercial and a gold-standard device), and three wristbands (a research-grade and two commercial devices). At least one or more devices include the following sensor data: oxygen consumption (VO2), fraction of oxygen in expired breath (FeO2), air moved by the lungs (Ve), volume breathed in a breath (Tv), breaths per minute (BR), humidity (H), temperature (T), pressure (P), acceleration (ACC), gyroscope (GYRO), photoplethysmography (PPG), electrocardiography (ECG), electrodermal activity (EDA), skin temperature (TEMP) and electroencephalography (EEG) and information derived from sensors such as e.g., heart rate (HR), heart rate variability (HRV), breathing rate (BR), body posture and more.

Table 1 presents an overview of existing datasets in the literature that enable EE modeling using sensor data. Only two of the existing datasets are publicly available for download, e.g.^3,9^, marked with “Yes” in the “Publicly Available” column of the table. In comparison to these datasets, our dataset contains a higher number of unique data sources (in total 18). Further, it is the only dataset that contains ACC and HR from multiple body locations, such as the ear, wrist, and chest, which allows researchers to investigate the development of novel techniques for EE estimation. Only Bouarfa *et al*.^10^ investigated the use of ACC placed on the ear to estimate EE. However, estimating EE from ACC and HR data collected from the ear has not yet been explored. Additionally, WEEE contains data from both medical grade devices (e.g., Zephyr Bioharness) and commercial devices (e.g., Fitbit sense and Apple Watch), which enables the comparison of HR measurements between such devices.

**Table 1:** Comparison of the existing datasets for energy expenditure modeling and our dataset.

Dataset Paper	Number of Subjects	Devices(s)	Sensor(s)	Body Location(s)	Number of Activities	Publicly Available
^46^	15	Polar S720i	HR	Wrist Chest	5	No
DEE^9^	28	Polar Active, DLW	HR, ACC	Wrist	(In-the-wild)	Yes
^10^	31	eAR, Cosmed K4b2	ACC VO2	Ear	10	No
^8^	24	Polar Active, DLW	ACC VO2	Wrist	2 (In-the-wild)	No
^47^	22	MetaMax 3B-R2, Polar H7	VO2, VCO2 ECG, HR, BR	Chest Upper body**	1*	No***
^19^	10	Cosmed K4b, Zephyr BioHarness, BodyMedia Fit	VO2 ECG, RESP, HR, BR, HRV ST, ACC, EDA, RR	Chest Thigh Arm	15	No
^48^	15	Cosmed K4b2, ECG Necklace	VO2 ACC, ECG, HR	Ankle Chest Thigh Wrist Waist	32	No
^2^	12	Cosmed K4b2, Smartphone	VO2 ACC	Waist	6	No
^49^	10	Cosmed K4b2, ECG Necklace	VO2 ACC, ECG, HR	Chest	41	No
4TU^50^	37	GeneActives Equivital, COSMED Activ8	ACC ACC, ECG,TEMP, HR, BR VO2, VCO2 ACC	Ankle Wrist Chest	16	No***
^6^	22	Oxycon Mobile Actigraph GT1M	VO2 ACC	Hip	22	No
JSI^3^	10	Shimmer, Zephyr BioHarness, BodyMedia FIT, Cosmed K4b2	ACC ECG, RESP, HR, BR, HRV ST, ACC, EDA, RR VO2	Arm Wrist Chest Thigh Ankle	15	Yes
*WEEE*	17	VO2 Analyzer, Nokia Earbuds, Empatica E4, Zephyr Bioharness, Wahoo Tickr, Apple Watch, Fitbit Sense, Muse S	VO2, FeO2, Ve, Tv, H, T, P ACC, GYRO, PPG EDA, TEMP, PPG, TEMP ECG, RESP, HR, BR, HRV HR, BR HR HR EEG, ACC, GYRO	Ear Chest Wrist Head	6	Yes

The table shows the dataset name and paper where it was presented, the number of subjects in the existing datasets, devices used, types of sensors and body locations at which sensors are placed during the data collection as well as number of physical activities. We linked the publicly avaialble dataset name to the repository where it can be downloaded. In this literature review we favored work that collected data from more than one data source or body location. For a more detailed overview of other existing datasets please refer to Alvarez *et al*.^3^.

*Cycling performed at different intensities. **In the form of a shirt. ***Available by sending a request to the corresponding author. HR - Heart rate, ACC–acceleration, VO2–oxygen consumption, VCO2–carbon dioxide exhaled, ECG–electrocardiography, BR–breathing rate, EDA–electrodermal activity, TEMP–skin temperature, HRV–heart rate variability, RR–Interbeat interval, FeO2–fraction of oxygen in expired breath, Ve–air moved by the lungs, Tv–volume breathed in a breath, BR–breaths per minute, H–humidity, T–temperature, P–pressure, GYRO–gyroscope, PPG–photoplethysmography, EEG–electroencephalography.

## Methods

To enable multimodal EE modeling, we design a controlled experiment and ask participants to perform a set of pre-defined activities. We opt for a controlled study because, despite its constraints, it enables running detailed analysis of the phenomenon under investigation and it is suitable for the replicability of the data collection procedure. In this section, we provide details about the participants, data collection setup and protocol, and the collected data.

### Participants

We recruited 17 participants (12 males and 5 females) using snowball sampling^11^. Participants were of age between 23 and 41 years old (MEAN = 30, STD = 5) and with an average BMI of 24.5 (STD = 2.9). The study was conducted following the ethical regulations at our institution. All the participants signed an informed consent form and agreed their data to be used for research purposes. Participants were instructed to wear comfortable attire for the experiment. Also, we asked participants to be in a rested and fasting state by refraining from endurance training for 24 hours prior the study and avoiding caffeine, tobacco, alcohol, and food intake 3 hours before the experiment. Participants were compensated with a £20.- gift card.

### Setup

**Fig. 1:**
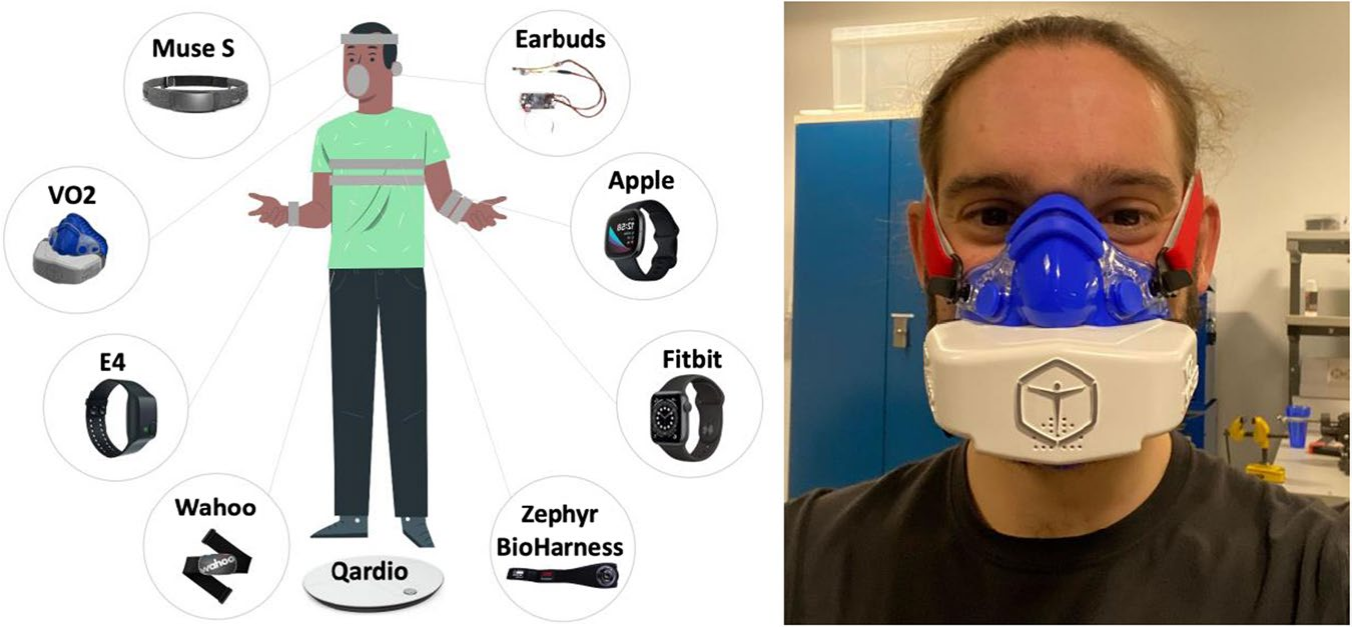
Study setup–Data collection setup (left) and a participant wearing the indirect calorimetry (right). We obtained consent from the participant to include in the manuscript the image on the right.

As a preparation for each experiment, we charged the devices and visually verified that the clock of each device matched the same time reference to ensure synchronization among the devices. This included checking for the date, time (in terms of hours, minutes and seconds) and time zone. Before the experiment, participants completed a set of questionnaires regarding their eating habits, sleep, stress and physical activity level. Before starting the experiment, we asked the participants to step up on the QardioBase smart scale (https://www.qardio.com/qardiobase-smart-scale-iphone-android/) to measure body composition metrics (e.g., weight, muscle percentage). We then placed the devices as follows on the participant: VO2 Master Analyzer (https://vo2master.com/) on the face, Nokia Bell Labs earbuds^12,13^ on the right ear, Muse S headband (https://choosemuse.com/muse-s/) on the head, Empatica E4 wristband^14^ on the non-dominant hand, Zephyr BioHarness chestbelt (https://www.zephyranywhere.com/) and Wahoo Tickr chest strap (https://eu.wahoofitness.com/devices/heart-rate-monitors) on the chest, Fitbit Sense watch (https://www.fitbit.com/global/us/products/smartwatches/sense) and Apple watch (https://www.apple.com/apple-watch-series-6/index.html) on the dominant hand. Figure 1 presents an overview of the study setup, devices used and their location. We ensured proper attachment of the face mask and calibration of the flow sensor, as recommended in^3^. Muse S headband, Zephyr Bioharness and Wahoo chestbelts were moisturized with water before attaching them to participant’s body. The earbuds are a multi-sensory earable device under development by the Nokia Bell Labs, which has been already tested in^12,13,15^. The VO2 Master Analyzer device has a smaller size than the major portable metabolic analyzer brands, which makes it a suitable option for VO2 measurements. Montoye *et al*.^16^ have shown acceptable validity and reliability of this device in comparison to gold-standard measurements. Furthermore, the VO2 Master Analyzer is compatible with other devices, such as, e.g., the Wahoo Tickr– validated in^17^–, which makes it easier for the researchers to obtain additional data (e.g., heart rate) together with VO2 measurements. The Zephyr BioHarness chest belt contains an ECG sensor, which provides heart rate measurements. Nazari *et al*.^18^ have shown evidence of the reliability and validity of heart rate measurements across multiple contexts using this device. The Zephyr BioHarness has been used also in other studies^3,19–21^. The Empatica E4 device is a watch-like, multi-sensor device. It is light, easy to use and comfortable to wear, which makes it suitable to monitor people’s energy expenditure. Additionally, the Empatica E4 provides the raw sensor data as well as encrypts the data during transfer and does not store user’s personal data, which is convenient to preserve the privacy of the study participants. The Empatica E4 has been extensively used in the literature for energy expenditure estimation^21^, but also other tasks^15,22,23^. We chose the Fitbit and Apple watch devices because they are among the most popular smartwatches available in the market, as shown in a recent article by *The Economist* magazine in^24^. Also, they have shown high accuracy for measuring heart rate during physical activities considered in our work (e.g., cycling, running)^25^. We chose the Muse S device because it is a portable and unobtrusive brain-sensing headband and has been previously validated in the literature^15,26,27^.

### Procedure

**Fig. 2:**
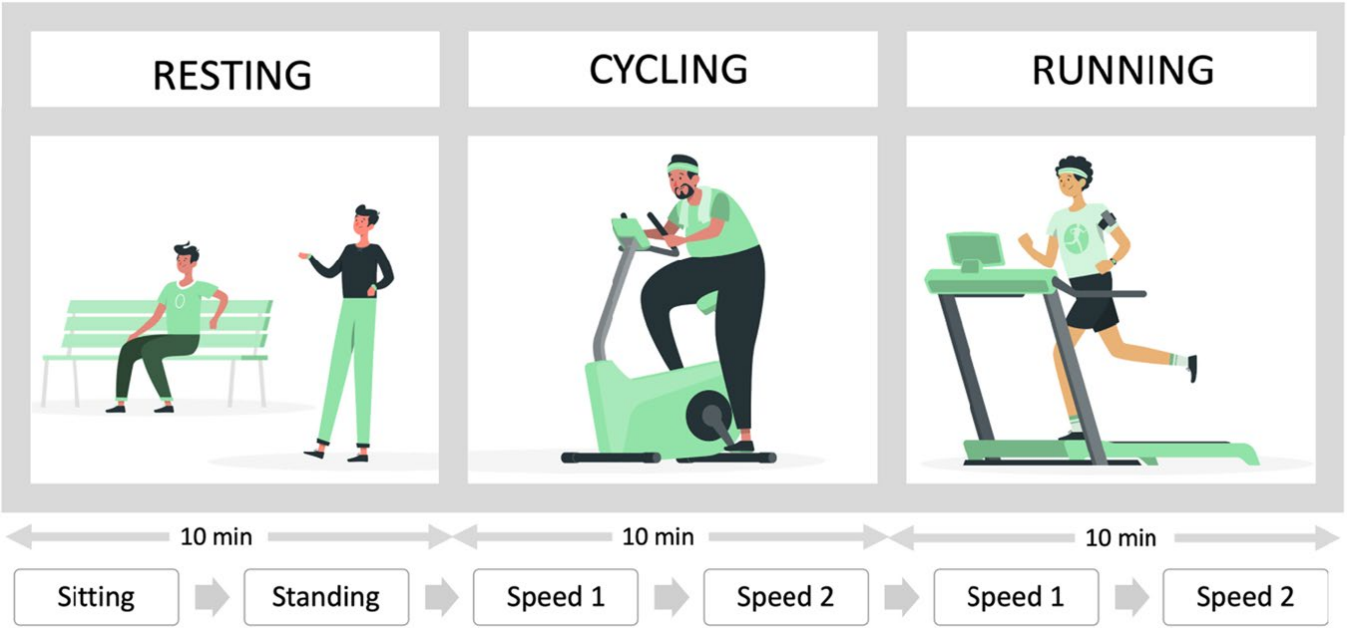
Data collection protocol. Speed 1 and Speed 2 during cycling and running refer to low and high speed levels. Such levels were chosen by the individual based on their fitness level. The average speed during the low and high levels of cycling was 15.64 (STD = 3.33) and 24.17 (STD = 4.94), and for running was 5.93 (STD = 1.48) and 8.58 (STD = 2.55).

Figure 2 depicts an overview of the study protocol. Participants followed a predefined set of activities, similar to^28,29^, grouped into three parts: *resting*, *cycling* and *running*. During resting, participants were asked to sit on a chair and stand on their feet, for 5 minutes each, to obtain physiological data during a resting state. After that, they cycled in an indoor bike and run on a treadmill, for 10 minutes in each activity. Both cycling and running activity were performed in two intensity levels, each of 5 minutes. We used a window of 5 minutes for each activity to reach a steady state EE, as recommended in^3^. The intensities of these activities were selected by the participants to represent their individual habits, as suggested in previous work^30,31^. The total duration of the experiment was 30 minutes. For consistency, the bicycle resistance and treadmill inclination were kept the same for all participants.

We picked resting, cycling and running activities because these activities involve movements of different intensity levels (e.g., light, moderate and vigorous). For instance, sitting or standing requires no or light movement, cycling requires half-body or moderate movement and running full body or vigorous movement. We run the protocol from low to high intensity to avoid the impact of high activities into low intensity ones.

### Collected data

We collect five types of data: *sensor data, respiratory gases, demographics and body composition, activity data and questionnaire*s data explained as follows.

#### Sensor and respiratory gases

Table 2 shows an overview of the characteristics of devices used to collect *WEEE* dataset. The table presents the device used, device location, the type of data that was collected for each device as well as paper(s) that validated the sensor readings of the device. The table shows that WEEE contains data from 8 different devices (including an indirect calorimeter serving as ground-truth information) placed on 5 unique body locations. Some of the sensors (e.g., ACC, PPG) are available in more than one body location (e.g., ear, wrist, chest).

**Table 2:** Overview of the devices used to collect our dataset, the body location of the device, types of sensors, measurement unit and sampling frequency as well as existing papers that validated the device.

Device	Position	Sensor(s)	Sampling	Unit	References
VO2 Master Analyzer face mask	Mouth	Oxygen consumption (VO2), Fraction of oxygen in expired breath (FeO2), Air moved by the lungs (Ve), Volume breathed in a breath (Tv), Breaths per minute (Rf), Humidity (H), Temperature (T), Pressure (P)	1 Hz	mL/kg/min, mL/min % L/min, L, BPM, %RH, C, hPa	^16^
Nokia Bell Labs earbuds	Ear	Accelerometer, Gyroscope, Photoplethysmography (Green, infrared, and red channels)	100 Hz	milli-g [−2000, +2000], milli-dps [−500000, +500000]	^12,13,15^
Muse S headband	Head	Accelerometer, Gyroscope, Electroencephalography (EEG) raw, EEG absolute band power (alpha, beta, delta, gamma, theta)	52 Hz, 52 Hz, 256 Hz, 10 Hz	g [−2:+2], deg/s [−245:+245], uV [0.0:1682.815], Bels	^15,26,27^
Zephyr BioHarness chest-belt	Chest	Accelerometer, Breathing sensor raw output, Breathing rate, Breath-to-breath interval, ECG raw waveform, Heart rate, Heart rate variability, RR interval, Posture	100 Hz, 25 Hz, 1 Hz, -, 250 Hz, 1 Hz, 1 Hz, -, 1 Hz	bits {0–4094}, bits {1:16777215}, bpm [4:70], ms, bits {0:4095}, bpm [25:240], ms {0:65534}, ms {0:32767}, Degrees from vertical {−180:180}	^3,18–21^
Wahoo Tickr chest strap	Chest	Heart Rate, Respiration Rate	1 Hz	bpm	^17^
Empatica E4 wristband	Wrist	Accelerometer, Blood Volume Pulse, Electrodermal activity, Skin temperature	32 Hz, 64 Hz, 4 Hz, 4 Hz	g {−2:+2}, -, microsiemens, C	^14,15,21,22,51^
Fitbit Sense Watch	Wrist	Heart Rate	Every 5–15 s	bpm	^25^
Apple Watch Series 6	Wrist	Heart Rate	1 Hz	bpm	^25^

#### Demographics and body composition

To collect body composition and demographics data, we use QardioBase smart scale. In particular, we collect participants’ gender, age, height, weight, percentage of body fat, muscle, bone, water and body mass index (BMI). Muscle mass percentage is calculated as the percentage of muscle in the body as compared to total body weight. Table 3 shows the mean (standard deviation) of the demographics and body composition data for all participants as well as for participants with female or male body types. The range of BMI is 20 to 30 kg/m^2^ (MEAN = 24.5, STD = 2.9).

**Table 3:** Mean (standard deviation) of the demographics and body composition data of the participants in our dataset.

	All	Male	Female
Participants	17	12	5
Age	30.2 (5.5)	30.7 (5.9)	28.8 (4.4)
Height (cm)	172.2 (6.9)	175.2 (5.4)	165.0 (4.5)
Weight (kg)	72.9 (11.6)	77.6 (9.8)	61.5 (6.9)
BMI (kg/m^2^)	24.5 (2.9)	25.4 (2.7)	22.4 (2.2)
Muscle (%)	14.8 (1.1)	14.7 (1.2)	14.8 (0.8)
Fat (%)	22.2 (5.8)	20.0 (4.9)	27.4 (4.4)
Bone (%)	4.2 (0.4)	4.2 (0.4)	4.2 (0.4)
Water (%)	52.2 (4.0)	52.5 (4.3)	51.6 (3.5)

#### Activity data

We derive labels regarding the activity performed from the protocol. Also, we kept notes of the intensity level (speed) of each activity. To enable further comparisons, we include the metabolic equivalent of a task (MET) values for each activity type based on intensity as defined in the compendium of physical activities^32^.

#### Questionnaires

We assess participant’s physiological and physical state before the experiment using validated questionnaires. In particular, we evaluate their sleep quality level over the past month using the *Pittsburgh Sleep Quality Index (PSQI)*^33^ and sleepiness level before the experiment using the *Stanford Sleepiness Scale (SSS)*^34^. Participants also report their stress level using the *Perceived Stress Scale (PSS)*^35^, physical activity level using the *International Fitness Scale (IFIS)*^36^, the readiness for physical activity using the *Physical Activity Readiness (PAR-Q)*^37^, and *How healthy is your diet?* to measure the nutritional value of their diet, which have an impact on EE.

## Data Records

The raw data can be found at Zenodo^38^ and the dataset is available for download at this link: 10.5281/zenodo.6420886. Data of each participant has been anonymized with an alphanumeric format of P#, to which we refer to as participant identifier, and is placed on separate folders named with participant identifier (e.g., P1). The dataset contains a folder for each participant and some other files described as follows: *Demographics.csv* contains demographics (e.g., gender, age) and body composition data (e.g., BMI, percentage of fat, muscle, water, bone) for each participant in an anonymous format, *Study_Information.csv* contains the start and end time of each study condition (e.g., start time of the sitting or cycling activity), speed of cycling/running and MET information for each activity, *Questionnaires* folder contains the answers to the pre-study questionnaires regarding participants’ physiological state. Within each participant folder, there are five other folders, namely, *VO2*, *EARBUDS*, *E4*, *ZEPHYR*, and *MUSE*, which contain the raw data obtained from each device during data collection. Table 4 provides an overview and description of the main files inside a participant folder.

**Table 4:** Description of the content of the folders named P# in the WEEE dataset. In this table we explain only the most relevant files in the dataset. The files inside the VO2 folder contain also the data collected from the Wahoo Tickr chest strap.

Device	File	Column(s)	Description
VO2	*DataAverage.csv*	Time[s]	Seconds (s) passed since the start of the session.
		Time[hh:mm:ss]	Time in hours:minutes:seconds format.
		VO2[mL/kg/min]	Oxygen consumption in mili-liter per kilogram per minute.
		VO2[mL/min]	Oxygen consumption in mili-liter per minute.
		HR[bpm]	Heart rate measured using the Wahoo Tickr chest strap.
		Rf[bpm]	Breaths per minute measured using the Wahoo Ticker chest strap.
		Tv[L]	Volume breathed in a breath.
		Ve[L/min]	Air moved by the lungs.
		Ve/VO2	PPG sensor green wavelength.
		FeO2[%]	Fraction of oxygen in expired breath.
		Pressure[hPa]	Pressure
		Temp[C]	Temperature
		HUM[%RH]	Humidity
		RR[ms]	The time elapsed between two successive R-waves of the QRS signal
		Time	The date and time the sample was captured.
E4		Column 1	X-axis of accelerometer sensor.
	*ACC.csv*	Column 2	Y-axis of accelerometer sensor.
		Column 3	Z-axis of accelerometer sensor.
	*BVP.csv*	Column 1	Data from photoplethysmograph (PPG) sensor.
	*EDA.csv*	Column 1	Electrodermal activity expressed in microsiemens (S).
	*HR.csv*	Column 1	Average heart rate extracted from the BVP signal.
	*IBI.csv*	Column 1	The time of the detected inter-beat interval expressed in seconds (s).
	*TEMP.csv*	Column 2	The distance of the current beat from the previous beat in seconds (s).
	*info.txt*	Column 1	Skin temperature expressed in degrees on the Celsius (°C) scale.
			Further information regarding each csv file in E4 folder.
EARBUDS		Timestamp	Timestamp in UNIX format with millisecond resolution.
	*-imu-*.csv	ax/gx	X-axis of accelerometer/gyroscope sensor.
		ay/gy	Y-axis of accelerometer/gyroscope sensor.
		az/gz	Z-axis of accelerometer/gyroscope sensor.
	*-ppg-*.csv	timestamp	Timestamp in UNIX format.
		green	PPG sensor green wavelength.
		ir	PPG sensor infrared wavelength.
		red	PPG sensor red wavelength.
MUSE		Column 1	Timestamp in UNIX format.
	*acc.csv*	Column 2	X-axis of accelerometer/gyroscope sensor.
	*gyro.csv*	Column 3	Y-axis of accelerometer/gyroscope sensor.
		Column 4	Z-axis of accelerometer/gyroscope sensor.
	*eeg.csv*	Column 1	Timestamp in UNIX format.
		Column 2–6	EEG channels.
ZEPHYR	*_Summary.csv	Time[s]	The date and time the sample was captured.
		HR	Heart rate measured from the ECG sensor.
		BR	Breathing rate measured from a pressure sensor in the strap.
		SkinTemp	Skin temperature.
		Posture	Posture: 0° = subject vertical, 90° = subject prone, −90° = subject supine, ±180° = subject inverted.
		Activity	Vector magnitude of the three axial acceleration magnitudes over the previous 1 second, sampled at 100 Hz.
		PeakAccel	Peak acceleration magnitude from the previous second.
		BatteryVolts	Device battery: fully charged ~4.2 V and fully discharged ~3.6 V.
		BRAmplitude	Breathing rate amplitude used for internal development only.
		HRV	Heart rate variability.
		ECGAmplitude	Uncalibrated ECG amplitude measured from peak of the R wave to peak of the S wave of the QRS complex.

### Missing data

The MUSE S device data of participant P02 is missing due to a malfunction in the streaming of the sensor data to the third-party app MindMonitor (https://mind-monitor.com/), which we used to collect the data. Part of the VO2 data of P03 and P12 during the cycling condition and of P16 during the running condition was lost due to issues with the indirect calorimeter VO2 sensor.

## Technical Validation

We evaluate the technical validity of the dataset, i.e., whether the sensor measure what they are expected, in three ways: (1) by providing descriptive statistics of the data in comparison to the device manuals, (2) by investigating the relationship between physiological signals collected from different body locations and (3) comparing the changes in sensor data for different physical activities, as suggested in^39^.

Table 5 presents descriptive statistics of the collected data for each device together with reference values obtained from the devices’ manuals. These statistics support the validity of the dataset because the minimum and maximum values obtained from the sensors are within the expected range for the majority of the sensors. For instance, the minimum (47) and maximum (209) HR values measured with ZEPHYR are inside the expected range of [25:240]. Similarly, the minimum (0.87) and maximum (1.91) values of ACC sensor measured with the E4 devices are within the ±2 range. These observations confirm that the data in WEEE dataset are as expected according to the devices’ manuals. We observe that the minimum HR derived from the E4 and earbuds fall below the expected minimum, this could be due to the presence of motion artifacts in PPG signal from which HR is derived. We recommend careful identification and removal of artifacts in the PPG signal before further analysis.

**Table 5:** Descriptive statistics of the WEEE dataset.

	MEAN	STD	MIN	25%	50%	75%	MAX	Ref.
EE[Kcal/min]	3.59	2.82	0.44	1.16	2.72	5.26	17.59	—
WAHOO_HR[bpm]	102.92	28.32	36.00	83.00	99.00	123.00	195.00	[25, 240]
WAHOO_Rf[bpm]	22.09	8.21	0.00	16.29	20.91	27.34	54.00	[4, 70]
ZEPHYR_HR	98.01	25.99	47.00	81.00	94.00	114.00	209.00	[25, 240]
ZEPHYR_BR	19.59	5.94	7.00	15.00	19.00	24.00	37.00	[4, 70]
ZEPHYR_Posture	4.82	33.42	−169.00	−9.00	−3.00	24.00	171.00	[−180, +180]
ZEPHYR_Activity	0.21	0.29	0.00	0.01	0.07	0.28	1.29	[0, 16]
ZEPHYR_PeakAccel	0.46	0.69	0.01	0.04	0.15	0.55	5.48	[0, 16]
E4_ACC	1.02	0.10	0.87	0.98	0.99	1.01	1.91	[−2g, +2 g]
E4_EDA	2.65	5.12	0.04	0.33	0.48	2.42	44.54	[0.01 - 100]
E4_TEMP	33.47	1.09	30.71	32.87	33.31	34.07	36.77	[−40, +115]
E4_HR	70.82	26.20	2.60	52.76	73.14	88.49	186.92	[25, 240]
EARBUDS_HR	66.73	43.30	0.06	33.80	71.84	93.18	182.36	[25, 240]
EARBUDS_ACC	1000.20	34.72	855.78	987.59	991.90	1000.95	1262.22	[−2k, +2k]
EARBUDS_GYRO	17996.31	18599.98	200.32	1388.59	12156.08	28716.58	218536.08	[−50k, +50k]
MUSE_ACC	0.02	0.00	0.00	0.02	0.02	0.02	0.03	[−2, +2]
MUSE_GYRO	18.99	15.44	4.56	8.35	12.40	25.48	249.91	[−245, +245]

The table includes mean (MEAN), minimum (MIN), standard deviation (STD), maximum (MAX) and their corresponding reference values (Ref.) The variations in the minimum and maximum values of the same sensor (e.g., ACC) from different devices are mainly attributed to body position heterogeneity.

**Fig. 3:**
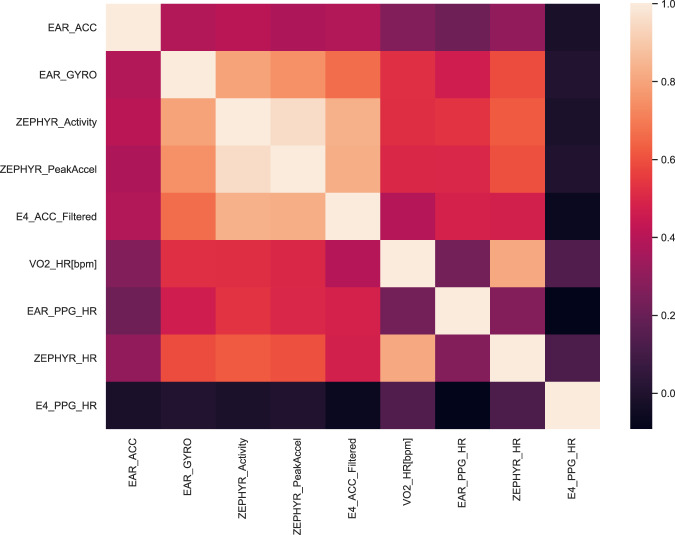
Correlation analysis–Correlation between physiological signals collected from different body positions.

To further evaluate the validity of our dataset, we explore the association between physiological signals collected from different body locations. Given that HR and ACC data are available from multiple body positions, we investigate the relationship between such data collected from different body positions. To perform this analysis, we compute Pearson product-moment correlation when data samples conform to a Gaussian distribution and Spearman rank correlation otherwise, as a common procedure in the literature^40^. We use Shapiro-Wilk test to verify whether the data conforms a Gaussian distribution. We test the p-values against both *p* < 0.05 threshold as well as the corrected threshold (${p}_{c}=\frac{p}{n}=0.01$, where *n* refers to body locations or devices and is equal to 5), to account for the Bonferroni correction^41^. Figure 3 presents the heatmap with correlations coefficients between sensor data collected from different devices. As expected, we observe that the motion data (e.g., ACC, GYRO) collected from the ear, chest or wrist is significantly positively correlated to each other (p < 0.01).

**Fig. 4:**
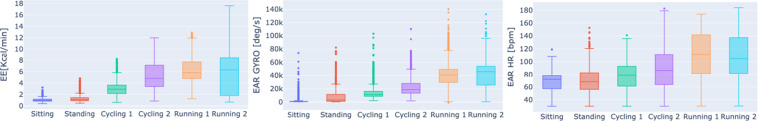
Data visualizations–Distribution of EE (left) measured using the indirect calorimetry, HR (middle) and GYRO (right) measured using the earbuds, during physical activities considered in this work.

We further explore the difference in sensor data for each physical activity. Figure 4 shows the distribution of EE measured using the indirect calorimetry (left), HR (middle) and GYRO (right) data measured using earbuds. As expected the average amount of EE during activities with high intensity movements is higher than for those with low intensity movements. For instance, the average EE during running or cycling are higher than during resting activities (e.g., sitting and standing). We observe similar patterns for HR and GYRO sensor data. This exploration of the data further confirms the validity and reliability of the collected data.

## Usage Notes

The WEEE dataset^38^ is available for download at this link: 10.5281/zenodo.6420886. The dataset website is https://wearableenergyexpenditure.github.io/. To analyze the dataset, we recommend using existing libraries for preprocessing and cleaning the physiological signals. In particular, the possible libraries that could be used are: HeartPy (https://python-heart-rate-analysis-toolkit.readthedocs.io/en/latest/) to extract heart rate data from PPG or ECG sensors, NeuroKit (https://neurokit2.readthedocs.io/en/latest/index.html) and BrainFlow (https://github.com/brainflow-dev/brainflow) to analyze EEG, PPG, ECG, and other kinds of data from physiological sensors available in the WEEE dataset, cvxEDA (https://github.com/lciti/cvxEDA) for decomposing the EDA signal into the phasic and tonic components, EDArtifact (https://github.com/shkurtagashi/EDArtifact) for exploring, preprocessing and identifying artifacts in EDA data and EDAExplorer (https://github.com/MITMediaLabAffectiveComputing/eda-explorer) to extract peaks from EDA signals and extract features from the ACC sensor.

The *WEEE* dataset fosters research and development of new solutions to problems as follows:

- *Device/Sensor Fusion*: The dataset contains raw measurements from sensors in multiple devices placed on the head, ear, wrist and chest. Thanks to its large number of wearable devices and sensor types, the dataset enables exploration of which sensor (device) or combination thereof enables a more accurate measurement of EE. For instance, the dataset enables exploring different sensor (device) fusion strategies such as e.g., stacking sensor channels one after the other, multi-input architecture, ensemble methods, and feature concatenation.
- *Sensor Location*: Researchers may further explore how the sensor position impacts the EEE. To the best of our knowledge, our dataset enables for the first time using heart rate and motion data collected from the ear for EEE and comparing it to the same data sources collected from other body positions.
- *Individual Characteristics*: The literature has shown that age, gender, body size and composition have an impact in EE. For instance, individuals with a larger body require a higher amount of energy than those with smaller body size because of the amount of tissues^4^. Our dataset enables a systematic, data-driven exploration of the impact of such individual characteristics in EEE.
- *Context Information*: Several researchers have shown that combining human activity recognition and EEE generally leads to better EEE^6^. Our dataset contains information about the type of activity that participants performed and its intensity level, which allows researchers investigating methods to simultaneously recognize the activity type, intensity level and EEE as well as understand their impact in EEE.
- *Physiological Conditions*: Investigating the impact of physiological conditions, e.g., physical activity level, diet, stress, and sleep in the overall EEE.
- *Data Quality*: Exploration of the impact of data quality (e.g., presence of noise and missing data) in the overall EEE. For instance, researchers could develop new methods to leverage the data from available sensors to handle noisy data, missing data points, missing sensor or device problems.
- *State-of-the-art Comparison*: The dataset also contains HR measurements from ECG sensor measured with Zephyr Bioharness and PPG sensor measured with research-grade devices (e.g., Empatica E4) and commercial devices (e.g., Fitbit and Apple Watch). This enables benchmarking existing HR-based EE measurement methods with new ones. Further, our dataset contains EE measurements from indirect calorimetry, which is one of the gold-standard measurement techniques for EE as well as METs derived from the compendium of physical activities based on activity type and intensity level. This enables the comparison of sensor-based EEE with gold-standard techniques.

While the WEEE data set opens up novel opportunities for computing systems that monitor energy expenditure, our approach presents some limitations and opportunities for further improvements. The first limitation stems from the low number of physical activities investigated. We opted for this decision to avoid having a long experiment protocol and to avoid causing fatigue to our study participants. Future work should consider extending our approach by adding more various physical activities. Even if our data set contains 3 activities, each of these activities has been performed in two intensity levels, which make the data set diverse in terms of types of activities and intensity levels.

### Indirect calorimetry data

The data collected from the indirect calorimetry can be used as a ground truth in future analysis. To prepare indirect calorimetry data for the analysis, the VO2 data should first be cleaned, for instance, by removing the values when VO2 sensor did not record any data (e.g., VO2 = 0). Then VO2 data should be converted to EE using equations from the literature e.g., in^4^.

### Earbuds data

To use the data collected from earbuds, one should first convert the raw ACC data to milli-g by multiplying it with 0.061 and the raw GYRO data to milli-dps (degrees per second) by multiplying with 17.5. This is to convert the raw data coming from the sensor from integer format to a more usable format (i.e., milli-g and milli-dps). Then remove the direct current (DC) offset from the GYRO data by applying a Butterworth band-pass filter. To clean the PPG signal, one could apply a Butterworth band-pass filter and then extract HR using the NeuroKit library mentioned before.

### Wristband data

To clean ACC and TEMP data, we suggest to apply a central moving average filter with a window of 1 minute, similar to^23^. Then to compute the ACC magnitude. The EDA data should be cleaned using a first order Butterworth low-pass filter with a cut-off frequency of 0.6 Hz, similar to^42^.  The EDA data can further be dicomposed into the *tonic*–the slowly changing component–and *phasic*–characterized by skin conductance responses (SCRs) or peaks that occur as a result of a stimuli–components, using the *cvxEDA* method proposed by Greco *et al*.^43^. To clean the PPG data, a first order Butterworth FIR filter with a cut-off frequency of 5 Hz  should be applied, as suggested in^44^. The HR data can then be derived from PPG using the NeuroKit library^45^.

### Questionnaire data

Figures 5 to 15 present a summary of the answers received from all the participants for the *PSQI*, *SSS*, *IFIS*, *PSS* and *“How healthy is your diet?”* questionnaires. Such data can be used as additional information regarding the physical and physiological state of participants before the experiment.

**Fig. 5:**
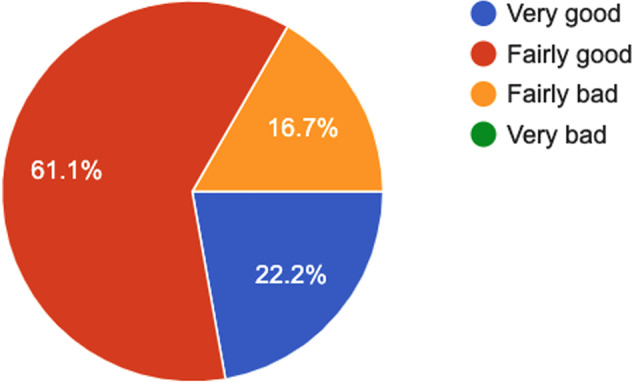
PSQI questionnaire^33^–Answers to the question “*During last week, how would you rate your sleep quality overall*?”.

**Fig. 6:**
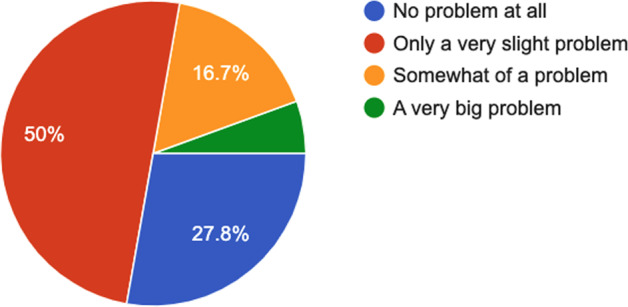
PSQI questionnaire–Answers to the question “*During last week, how much of a problem has it been for you to keep up enough enthusiasm to get things done?*”.

**Fig. 7:**
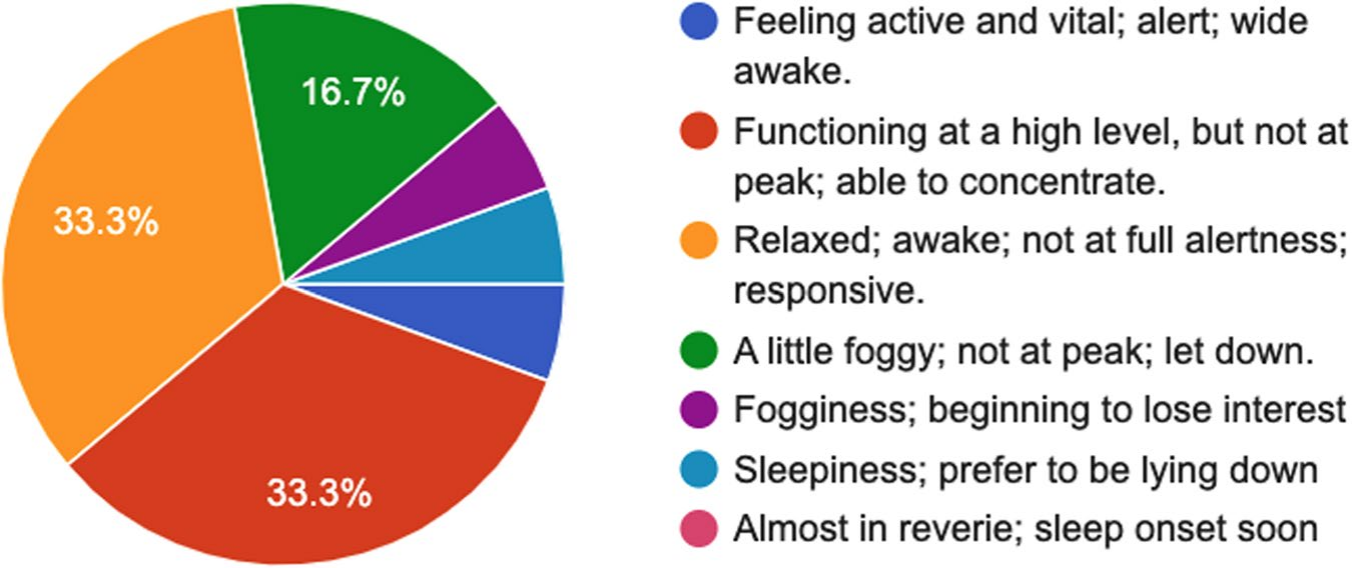
SSS questionnaire^34^–Answers to the question “*Please report your degree of sleepiness at the moment*”.

**Fig. 8:**
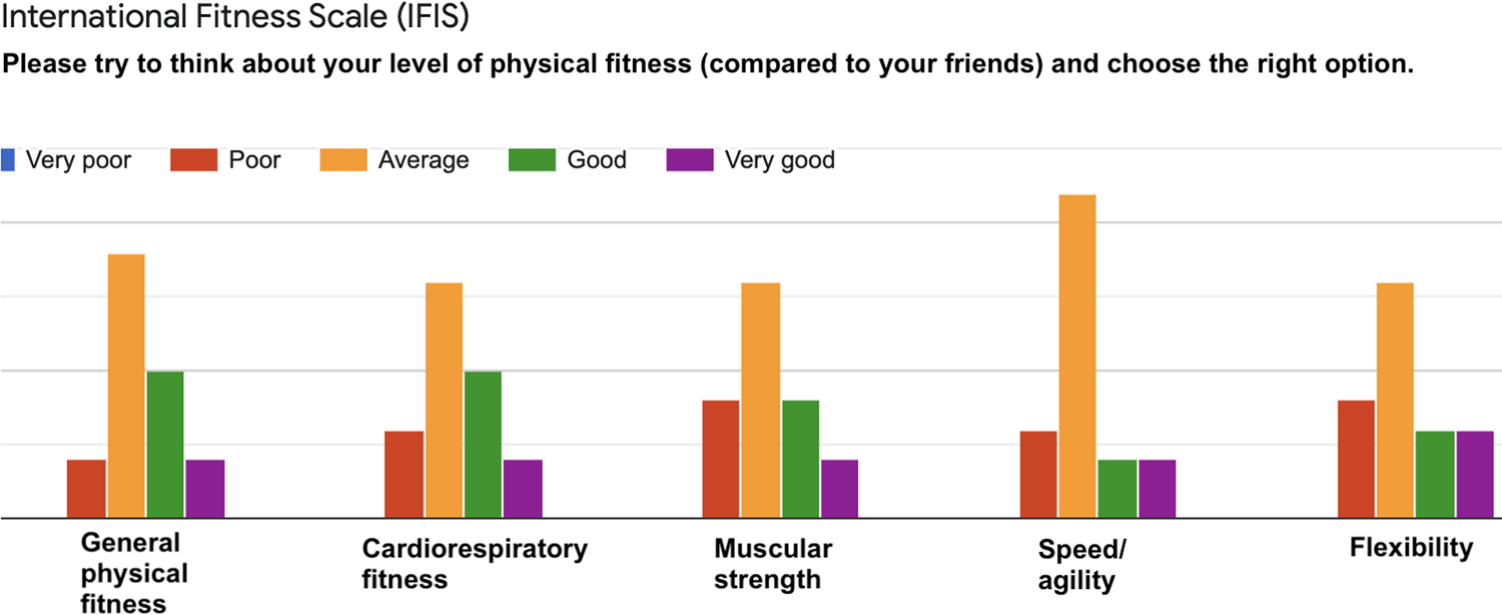
IFIS questionnaire^36^–Answers to the question “*Please try to think about your level of physical fitness (compared to your friends) and choose the right option*”.

**Fig. 9:**
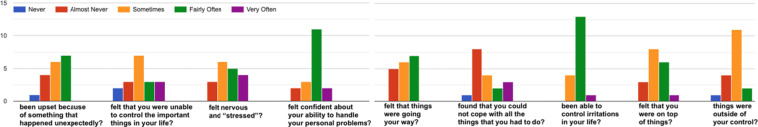
PSS questionnaire^35^–Answers to the question “*In the last week, how often have you…*”.

**Fig. 10:**
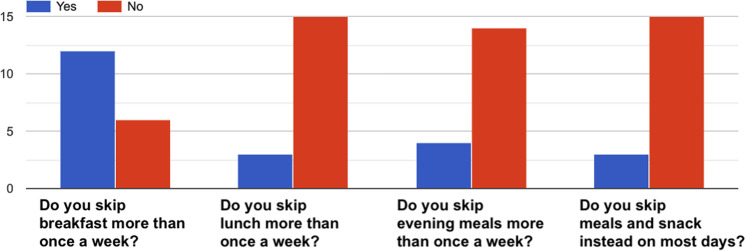
How healthy is your diet?^36^–Answers to the item “*Eating habits*”.

**Fig. 11:**
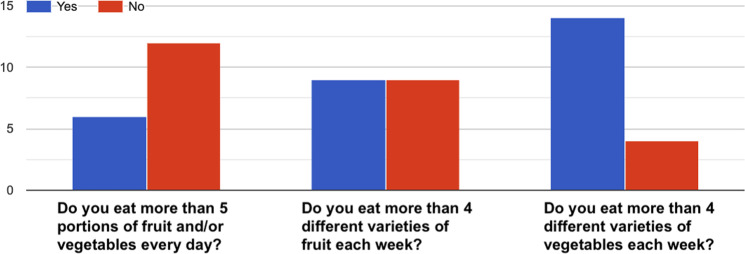
How healthy is your diet?^36^–Answers to the item “*Fruit and vegetables*”.

**Fig. 12:**
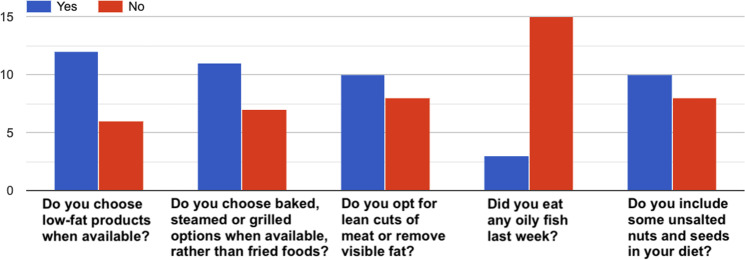
How healthy is your diet?^36^–Answers to the item “*Fat*”.

**Fig. 13:**
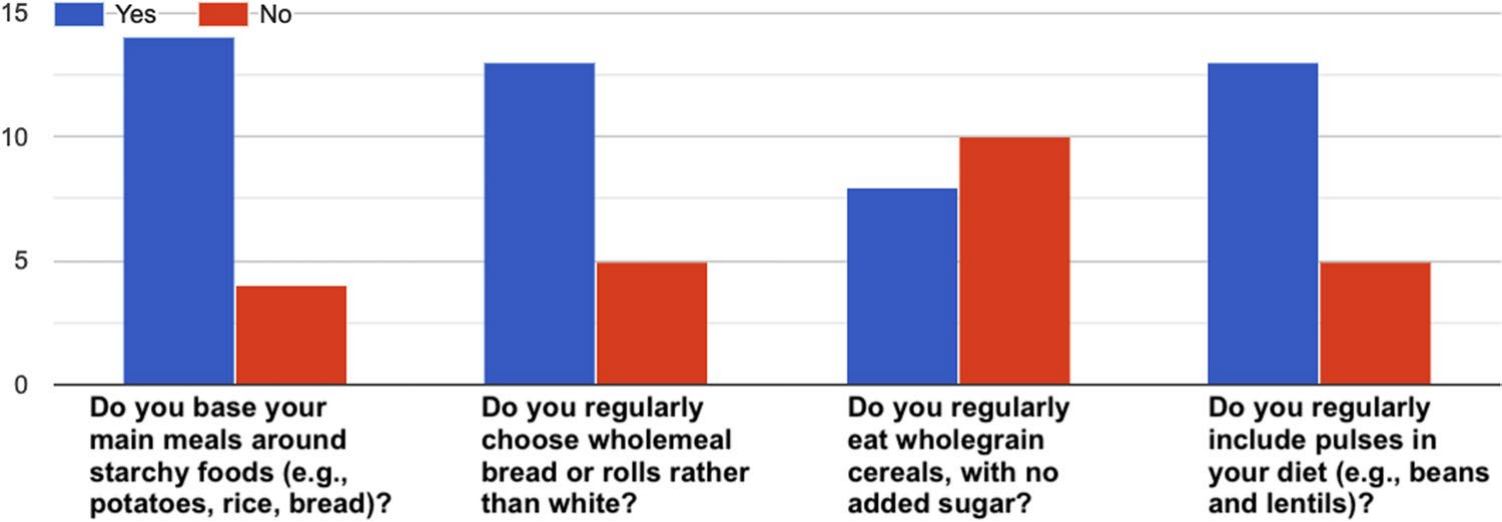
How healthy is your diet?^36^–Answers to the item “*Starchy foods*”.

**Fig. 14:**
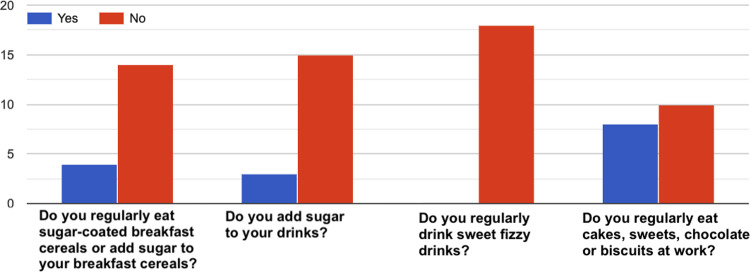
How healthy is your diet?^36^–Answers to the item “*Sugar*”.

**Fig. 15:**
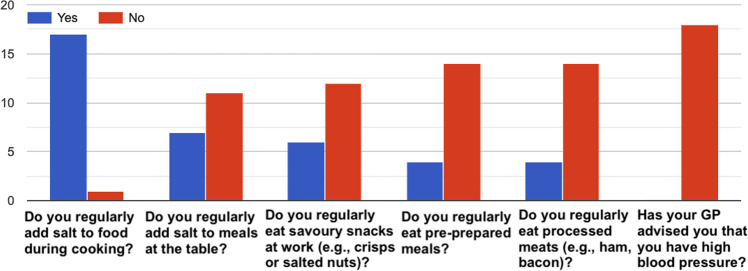
How healthy is your diet?^36^–Answers to the item “*Salt*”.

### Other data

The data from Wahoo Ticker and Zephyr BioHarness are preprocessed and provided at a 1 Hz granularity. For these reason, data from such devices can be used as is.

## Data Availability

We provide the raw csv data files obtained during the data collection structured by user and device identifier. We did not implement any custom code to generate or process the data.
